# Human thermoneutral zone and thermal comfort zone: effects of mild heat acclimation

**DOI:** 10.1186/2046-7648-4-S1-A7

**Published:** 2015-09-14

**Authors:** Hannah Pallubinsky, Lisje Schellen, Boris RM Kingma, Wouter D van Marken Lichtenbelt

**Affiliations:** 1 Department of Human Biology, NUTRIM, Maastricht University, The Netherlands; 2 School of Built Environment and Infrastructure, Avans University of Applied Sciences, The Netherlands

## Introduction

Overheating of buildings is currently a hot topic in the Western World. A significant amount of energy is needed for air conditioning and ventilation of public and commercial buildings. Nevertheless, up to 80% of the occupants are dissatisfied with the thermal environment, even though buildings meet thermal comfort criteria as determined by the ASHRAE Standard 55 and ISO Standard 7730. Physiological parameters such as sex, age, body composition and metabolic rate can have a great effect on an individual's perception of the thermal environment. A study among young Europeans indicated that the preferred ambient temperature might vary as much as 10 °C between individuals. However, it is not yet clear how an individual's thermal comfort zone (TCZ) relates to the physiological thermo-neutral zone (TNZ). Moreover, unlike thermoregulatory adaptations to strong repetitive heat challenges, it is unknown to what extent humans adapt to more mild warm ambient conditions in terms of subjective perception and thermo-regulatory physiology. Therefore, the present study aimed to investigate the relationship of an individual's TNZ and TCZ as well as the influence of 7 days of mild heat acclimation on TNZ and TCZ. Since the study is still ongoing, preliminary data will be presented.

## Methods

Twelve young, healthy males will visit the laboratory of Maastricht University for 10 consecutive days. At day 1 and 2, protocols 'neutral-to-warm' and 'neutral-to-cold' will be conducted. Both will consist of 60 minutes baseline at 30 °C, followed by a transient temperature change with 10 K.h to 40 °C for the 'neutral-to-warm' protocol and to 15 °C for the 'neutral-to-cold' protocol, respectively. Relative humidity will not be controlled in this setting. Participants will be situated in a climate chamber in supine position, wearing underwear only.

Mild heat acclimation (7 days) will start immediately after the 'neutral-to-cold' protocol and participants will be exposed to 34 °C for 6 h per day. At day 9 and 10, protocols 'neutral-to-warm' and 'neutral-to-cold' will be repeated. Energy expenditure will be measured by means of indirect calorimetry (Quark RMR, COSMED, Italy). Thermal comfort will be evaluated using a 7-point visual analogue scale ranging from -3 very uncomfortable to +3 very comfortable.

## Results

Preliminary data show that the protocols 'neutral to warm' and 'neutral to cold' allow for approximation of an individual's TNZ and TCZ. Energy expenditure data of the first participants shows inter-individual differences in basal metabolic rate, TNZ range and upper and lower critical temperatures. For the participants measured so far, prolonged exposure to mild heat (34°C, 6 h for 7 consecutive days) seemed to influence thermal perception and the TNZ. However, there is variation in response between individuals.

## Preliminary conclusion

The range and positioning of the human TNZ and TCZ vary among individuals. Prolonged mild heat exposure seems to be an effective way to extend or shift TCZ and TNZ. More data is needed to further elucidate the relationship between TNZ and TCZ. During the conference, definitive results of all subjects will be presented.

